# ERp29 as a regulator of Insulin biosynthesis

**DOI:** 10.1371/journal.pone.0233502

**Published:** 2020-05-20

**Authors:** Jeffrey Viviano, Margaret Brecker, Christine Ferrara-Cook, Laurence Suaud, Ronald C. Rubenstein

**Affiliations:** 1 Cystic Fibrosis Center, The Children’s Hospital of Philadelphia, Philadelphia, Pennsylvania, United States of America; 2 Department of Pediatrics, Perelman School of Medicine at the University of Pennsylvania, Philadelphia, Pennsylvania, United States of America; The University of British Columbia Life Sciences Institute, CANADA

## Abstract

The environment within the Endoplasmic Reticulum (ER) influences Insulin biogenesis. In particular, ER stress may contribute to the development of Type 2 Diabetes (T2D) and Cystic Fibrosis Related Diabetes (CFRD), where evidence of impaired Insulin processing, including elevated secreted Proinsulin/Insulin ratios, are observed. Our group has established the role of a novel ER chaperone ERp29 (ER protein of 29 kDa) in the biogenesis of the Epithelial Sodium Channel, ENaC. The biogenesis of Insulin and ENaC share may key features, including their potential association with COP II machinery, their cleavage into a more active form in the Golgi or later compartments, and their ability to bypass such cleavage and remain in a less active form. Given these similarities we hypothesized that ERp29 is a critical factor in promoting the efficient conversion of Proinsulin to Insulin. Here, we confirmed that Proinsulin associates with the COP II vesicle cargo recognition component, Sec24D. When Sec24D expression was decreased, we observed a corresponding decrease in whole cell Proinsulin levels. In addition, we found that Sec24D associates with ERp29 in co-precipitation experiments and that ERp29 associates with Proinsulin in co-precipitation experiments. When ERp29 was overexpressed, a corresponding increase in whole cell Proinsulin levels was observed, while depletion of ERp29 decreased whole cell Proinsulin levels. Together, these data suggest a potential role for ERp29 in regulating Insulin biosynthesis, perhaps in promoting the exit of Proinsulin from the ER via Sec24D/COPII vesicles.

## Introduction

The development of type 2 diabetes (T2D) is initially characterized by increased Insulin secretion by the pancreatic β cell to meet and overcome the demands of peripheral Insulin resistance. Eventually, the capacity of the β cell is reached, and frank diabetes occurs when there is an inability to secrete enough Insulin in response to glucose stimulation [1]. This chronic overstimulation leads to and is marked by Insulin secretory inefficiency and eventually β-cell demise [2]. β-cell Endoplasmic Reticulum (ER) stress [3, 4], which includes activation of the unfolded protein response (UPR), prevents the ER machinery from properly processing and exporting Proinsulin in response to an increased demand for Insulin biosynthesis and secretion. The failure to resolve the UPR ultimately leads to apoptosis of the β-cell [5]. Thus, it is clear that an ER-stressed β-cell would have increased difficulty facilitating proper Insulin biosynthesis. It is therefore critical to gain additional understanding of how resident chaperones of the ER may facilitate the folding and export of Proinsulin from the ER.

Insulin is first translated as Pre-Proinsulin in β-cells. Its signal peptide, or presequence, is cleaved in the ER, converting Pre-Proinsulin into Proinsulin [6]. Proinsulin is then transported from the ER to the cis-Golgi, likely via Coat Complex II (COP II) machinery [7]. It then transverses the Golgi and is packaged into secretory vesicles in the trans-Golgi. In these latter compartments it can undergo cleavage by furin-like convertases and carboxyendopeptidase E to form mature Insulin and C-peptide [8, 9]. Currently, the mechanism by which Proinsulin, which is presumably soluble in the ER lumen, is directed for inclusion in COP II ER→Golgi transport vesicles is not clear.

Interestingly, Proinsulin can bypass proper cleavage in the Golgi or later compartments to be secreted in its immature form as Proinsulin. In fact, an increased ratio of secreted Proinsulin/Insulin predicts future risk of T2D in otherwise “healthy” adults and is also seen in patients with Cystic Fibrosis (CF) [10], who have a 50% lifetime risk of developing Cystic Fibrosis Related Diabetes (CFRD). However, it is currently not known why this aberrant processing and increased secretion of Proinsulin portends the onset of T2D [11, 12]. It is therefore logical to examine the interacting partners of Proinsulin in the ER, as these proteins are likely critical in initially directing Proinsulin to the Golgi for proper maturation during processing.

Sec24 is a cytosolic protein and one of the five key components of the pre-budding complex that initiates COP II vesicle formation [13, 14]. It is recruited by the Sar1 GTPase to the ER membrane in a heteroduplex with Sec23 [14, 15]. This complex then binds Sec13/31, which is thought to induce deformation of the ER membrane and vesicle bud formation [13, 16]. These components of the COP II pre-complex in the cytosol are suggested to recruit both integral membrane cargo and membrane-associated proteins within the ER lumen [17, 18]. In particular, Proinsulin appears to be captured into COP II vesicles for transport from ER to Golgi in a manner that is Sar1-dependent; namely, when Sar1 is inhibited, Proinsulin is not cleaved into mature Insulin and C-peptide [19]. Our group previously demonstrated that the pre-budding complex component, Sec24D, plays a critical role in the trafficking and maturation of the epithelial channel, ENaC, and that it does this in concert with the ER resident protein and chaperone, ERp29 [20, 21].

ERp29 is a 29 kDa ER-luminal molecular chaperone that is ubiquitously expressed in mammalian tissues [22]. It is highly expressed in secretory cells, including islet β-cells, and has been shown to facilitate ER→Golgi transport of the sulfotransferase protein product of the *D*. *melanogaster* PIPE gene, which contributes to the coordination of the dorsal-ventral axis during *Drosophila* embryogenesis [23]. Furthermore, Muscle IGF-I receptor (IGF-IR)-lysine-arginine (MKR) diabetic mice express higher levels of ERp29 in their islet β-cells than controls [24]. Previous work from our group has demonstrated that ERp29 mRNA expression and protein abundance is increased by ~50% in epithelial cells with the addition of Sodium 4-Phenylbutyrate (4PBA) [25], a drug that our group has also demonstrated to correct the trafficking of the most common CFTR mutation, F508del [26]. In fact, we found that overexpression of ERp29 itself, without 4PBA treatment, can correct F508del trafficking [25]. ERp29 is hypothesized to interact with either Y/F-X-Y/F or Y/F-Y/F motifs on client proteins, and Pre-Proinsulin has one such motif, ^48^F-F-Y^50^ (^24^F-F-Y^26^ in Proinsulin). ERp29 has a single Cysteine (C157) that is not implicated in client interaction or in catalysis of client disulfide formation and exchange [27] and is extrinsic to its client binding site [28]. Our group’s data [21] suggest that mutation of this Cys to Ser (C157S) inhibits ERp29 function with regards to ENaC biogenesis, and interferes with the ion channel’s ability to interact with the Sec24D cargo recognition component of COP II.

ENaC biosynthesis in some ways parallels that of Proinsulin, in that both ENaC and Proinsulin are transported from the ER to Golgi, likely via COP II vesicles, and both are cleaved in the Golgi or later compartments by furin-like convertases into their respective mature forms [8, 9, 29]. Additionally both proteins can reach the surface (ENaC) or be secreted (Proinsulin) in their immature form, which may suggest the utilization of similar machinery and processes for maturation [29]. These considerations lead us to hypothesize that Sec24D and ERp29 also play key roles in Insulin biosynthesis and trafficking.

## Materials and methods

### Cell culture

Min-6 mouse insulinoma cells were purchased from AddexBio (C001808) and were maintained in AddexBio Optimized DMEM (C003-02), 15% FBS (Gemini), and 0.05mM 2-mercaptoethanol (Sigma Aldrich). Ins-1 rat insulinoma cells were purchased from AddexBio (C0018007) and were maintained in AddexBio Optimized RPMI-1640 (C0004-02) + 10% FBS (Gemini), and 0.05mM 2-mercaptoethanol (Sigma Aldrich). Due to the documented changes in ERp29 expression in Min-6 over passage number [30, 31], the experiments reported here were performed on passages 15–35 of non-glucose stimulated Min-6 cells.

### Depletion of Sec24D or ERp29 by siRNA

Sec24D or ERp29 expression was depleted in Min-6 cells using pools of specific small interfering RNA (siRNA) (Dharmacon/Thermo Fisher Scientific; L-065430-01 and L-061755-01, respectively). 20 pmol of specific siRNA or non-targeting control siRNA (D-001810-10 Dharmacon/Thermo Fisher Scientific) were delivered to Min-6 cells by transfection with Lipofectamine 2000 according to the manufacturer’s protocol. Cells were subsequently assayed 72 hours after transfection as described below.

### Transient overexpression of ERp29

pcDNA4 plasmids encoding wt ERp29 or ERp29 C157S [32] were used in these experiments. Plasmid maxi-preps were prepared using the QIAfilter Plasmid Maxi-Prep kit (Qiagen). Transient transfections of Min-6 were performed with Lipofectamine 2000 reagent (Invitrogen) according to the manufacturer’s protocol. Assays were performed 72 hours after transfection as described below.

### Antibodies

Rabbit anti-ERp29 (ab11420), mouse monoclonal anti-Proinsulin (ab8304), rabbit anti-Sec24D (ab191566), and mouse anti-GAPDH (ab8245) were purchased from Abcam (Cambridge, MA). Mouse monoclonal anti-Proinsulin was purchased from Cell Signaling Technologies (Danvers, MA, Catalog L6B10) and from Sigma-Aldrich (St. Louis, MO, Catalog SAB4200691). Rat monoclonal anti-C-peptide (NBP-05439) was purchased from Novus (Centennial, CO). Mouse anti-BiP (610979) was purchased from BD Biosciences (Franklin Lakes, NJ). Horseradish peroxidase-conjugated secondary antibodies (anti-mouse, NA931V and anti-rabbit, NA934V) were from GE Healthcare (Pittsburgh, PA).

### Co-immunoprecipitation

For co-immunoprecipitation experiments, Min-6 or Ins-1 cells were lysed under non-denaturing conditions in RIPA buffer without SDS, or using Thermo Lysis buffer, and protein content was determined using BioRad DC reagents. For immunoprecipitations, 10 μl of the indicated primary antibody was incubated with whole cell lysate proteins (500 μg total protein) at room temperature for 1 h, and then incubated with Protein G PLUS agarose beads (22811, Thermo Fisher Scientific, Waltham, MA) for 2 h. Unless otherwise specified, (Pro)Insulin immunoprecipitations were performed using the Mouse monoclonal anti-Proinsulin was purchased from Cell Signaling Technologies (Danvers, MA, Catalog L6B10). Precipitated proteins were released by heating the samples for 10 min at 90°C in 6X Laemmli sample buffer, resolved by SDS-PAGE and revealed by immunoblot as described below.

For analysis of co-immunoprecipitated proteins by ELISA, 2 μl primary antibody was incubated with cell lysate proteins (50 μg total protein) for 1 h and then incubated with Protein G agarose for 2 h at room temperature. Precipitated proteins were released by heating the samples for 30 min at 37°C in RIPA Lysis buffer with 0.1% SDS and analyzed by ELISA as described below.

### ELISA

Proinsulin (10-1232-01) and Insulin (10-1247-10) ELISA kits were purchased from Mercodia (Uppsala, Sweden) and were used according to manufacturer’s specifications. For Co-immunoprecipitations, precipitated Proinsulin was detected in 2 μl of the final eluate. For measurement of total cellular Proinsulin, cell lysate samples were diluted 1:1000 in 20 mM Tris pH 7.5, 150 mM NaCl. To measure total cellular Insulin levels, cell lysate samples were diluted 1:10,000 in 20 mM Tris pH 7.5, 150 mM NaCl. For analysis, the Proinsulin or Insulin content was normalized by the total protein content of the whole cell lysate.

### Immunoblot

Our general techniques for immunoblot analyses were published previously [21]. Briefly, whole cell lysates were prepared by incubating cells on ice for 30 min in RIPA buffer (150 mM NaCl, 50 mM Tris-HCl, pH 8, 1% Triton X-100, 1% sodium deoxycholate, 0.1% SDS) containing a 1:1000 dilution of protease inhibitor cocktail (Sigma-Aldrich), or using Pierce IP Lysis Buffer (Thermo Scientific, # 87788) according to the manufacturers specifications. The lysates were cleared by centrifugation (14,000 X g for 15 min at 4 °C). Protein content in the lysate supernatants was determined using DC protein assay reagents (Bio-Rad) and bovine serum albumin (BSA) as a standard.

Samples were denatured for 20 min at 95° C using 6X Laemmli sample buffer (125 mM Tris, pH 6.8, 4% SDS, 10% glycerol, 0.006% bromophenol blue, 1.8% 2-mercaptoethanol, final concentration 1-2X). Equal amounts of protein (typically 50 μg) were resolved using SDS-PAGE and then transferred to nitrocellulose (Bio-Rad Nitrocellulose membranes 0.2 μm Cat. #1620147) using semi-dry techniques (Bio-Rad). Nonspecific protein binding was diminished by incubating the membrane in 5% nonfat milk in TBS (10 mM Tris-HCl, pH 8, 150 mM NaCl) with 0.1% Tween 20. Primary antibodies and horseradish peroxidase-conjugated secondary antibodies were applied in TBS with 0.1% Tween 20 and 5% nonfat milk. Difficulty in the detection of Proinsulin and Insulin on these blots using single primary antibodies was remedied by optimizations that included using a combination of primary mouse monoclonal antibodies including anti-Insulin/Proinsulin from Sigma, Cell Signaling Technologies, and Abcam at a 1:1000 dilution for each antibody. Immunoreactivity was detected by chemiluminescence (SuperSignal West Pico or Femto; Thermo Fisher Scientific, Waltham, MA) and fluorography using either film (Hyblot ECL, Amersham) or a ChemiDoc Touch Imaging System (version 5.2.1; Bio-Rad, Hercules, CA). Densitometry was performed using the Image Lab Software (Bio-Rad) for images visualized by the ChemiDoc System.

### Recombinant protein standards

The following recombinant human proteins were used for the standardization of experiments and as positive controls for immunoblots: Human Proinsulin C-peptide Analogue Protein (NBP2-35211, Novus). Recombinant human Proinsulin (1336-PN, R&D systems). Insulin Recombinant protein (NBP-1-99193, Novus).

### Statistical analysis

Statistical significance was determined by a two-tailed Student’s t-test or with a Mann-Whitney U/Wilcoxon Rank Sum test if data were not normally distributed. For immunoblot data, densitometry of the experimental lanes are expressed relative to the densitometry of their respective controls, and a Wilcoxon signed-rank test was utilized to test for differences from the reference value of 1.0. Graphs of data were generated and statistical analysis was performed using Prism GraphPad v7.04; these graphs depict individual data points as well as means +/- SEM. A p-value of ≤0.05 was considered significant.

## Results

### Optimization of Proinsulin and Insulin detection methods

**Fig 1:**
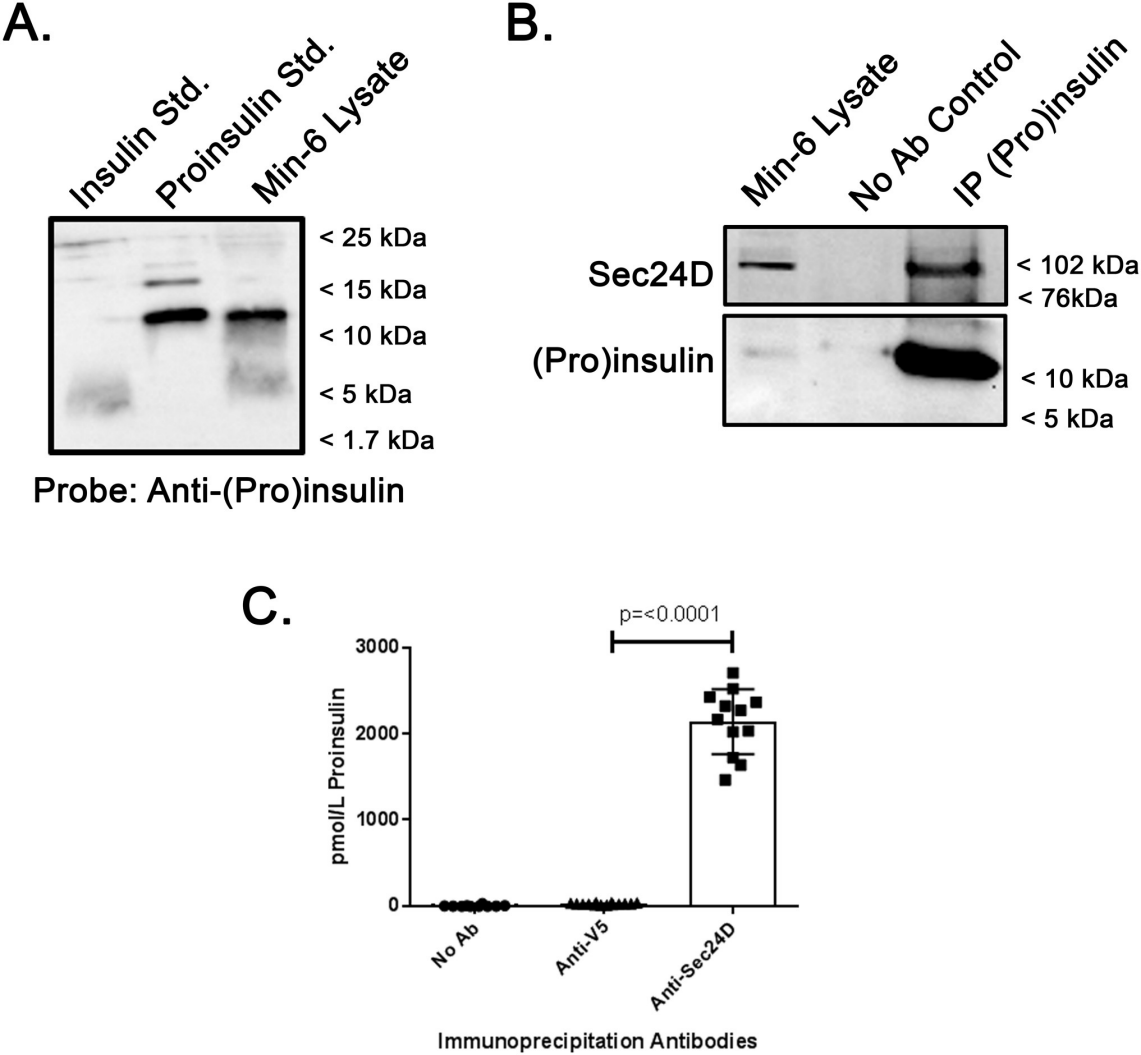
Proinsulin interacts with the COP II cargo recognition component Sec24D. (A) Purified recombinant human Insulin, purified human Proinsulin, and whole cell lysate of Min-6 mouse insulinoma cells were resolved by SDS-PAGE and analyzed by immunoblot. (B) Min-6 cells were lysed under non-denaturing conditions and 500 μg of lysate was subject to immunoprecipitation with anti-Insulin/Proinsulin. Precipitated proteins were resolved by SDS-PAGE and immunoblots were probed for Sec24D or Proinsulin as indicated. The Min-6 lysate lane was loaded with 50 μg (10%) of the input lysate. (C) Min-6 cells were lysed under non-denaturing conditions and 50 μg of lysate was subject to immunoprecipitation with a no antibody control (n = 12), anti-V5 (non-specific antibody control, n = 14), or anti-Sec24D antibody (n = 12). Precipitated Proinsulin was quantified with ELISA as described in the materials and methods. Precipitated Proinsulin in anti-V5 Control vs anti-Sec24D, p = <0.0001.

To establish a baseline for the analyses of the interactions of Proinsulin and Insulin with trafficking machinery, human recombinant Proinsulin and Insulin standards, as well as Min-6 whole cell lysates were analyzed by immunoblot (Fig 1A). Here we observe a ~12 kDa species consistent with native Proinsulin and a ~6 kDa species consistent with native Insulin in our Min-6 Lysate when these blots were probed with a mixture of three different mouse Insulin/Proinsulin monoclonal antibodies. Through protocol optimization, we achieved the clearest, most distinct banding patterns using this three-antibody cocktail, perhaps due to variation in their respective targeted epitopes. The banding patterns of these Proinsulin and Insulin standards were then used as a reference for subsequent experiments. Furthermore, because these antibodies more robustly and consistently detected Proinsulin compared to Insulin, immunoblot analyses were primarily used to identify Proinsulin in the subsequent experiments. ELISAs were also used to quantify Proinsulin and Insulin to supplement our immunoblot observations and quantifications, and the specificity of these assays were independently confirmed (S1 Fig).

### Proinsulin interacts with the COPII cargo recognition component Sec24D

Previously, inhibition of Sar1 was used to demonstrate the role of COP II vesicle formation in the forward trafficking of Proinsulin [19]. Sar1 initiates the formation of the COP II pre-budding complex and recruits the Sec24/Sec23 heteroduplex to the ER membrane [14, 15]. Sec24D, a human isoform of Sec24, serves as a cargo recognition component for the COP II pre-complex. We therefore chose to investigate a potential role for Sec24D in directing Proinsulin to the Golgi for processing. Min-6 mouse insulinoma cells were lysed under non-denaturing conditions, and this lysate was subjected to immunoprecipitation with an anti-Insulin/Proinsulin antibody (Fig 1B). Here we observed that Sec24D co-precipitates with the Insulin/Proinsulin antibody, but did not precipitate when the primary antibody was omitted from the immunoprecipitation reaction. These data suggest an interaction between Proinsulin and Sec24D, either directly or, more likely, within a larger complex. To further assess the interaction of Sec24D and Proinsulin, the reciprocal experiment was performed, comparing either a no antibody control or anti-V5 (as a non-specific antibody control) with immunoprecipitation using anti-Sec24D. Precipitated Proinsulin was then quantified with ELISA (Fig 1C). These data demonstrate that Proinsulin co-precipitates only in the presence of anti-Sec24D. Proinsulin did not precipitate in the no antibody control or with anti-V5 (non-specific). These data suggest that Proinsulin precipitation is the result of an interaction with Sec24D, likely as part of a larger complex, and is not due to non-specific precipitation (anti-V5 Control vs anti-Sec24D, p = <0.0001).

To confirm that this observation is not a cell-model- or species-specific phenomena, similar experiments were performed in Ins-1 rat insulinoma cells. When immunoprecipitation was performed with anti-Insulin/Proinsulin, we again observed that Sec24D was present in precipitated protein by immunoblot (S2A Fig). Proinsulin was also detected in precipitated proteins with ELISA when immunoprecipitation was performed using anti-Sec24D, but was not detected in either the no antibody control or the anti-V5 (non-specific) control (S2B Fig, anti-V5 Control vs anti-Sec24D p = <0.0001). Together, these data suggest that Sec24D interacts with Proinsulin, either directly or, more likely, within a larger complex, in a manner that does not appear to be species- or cell-model- specific.

### Sec24D regulates intracellular Proinsulin abundance

**Fig 2:**
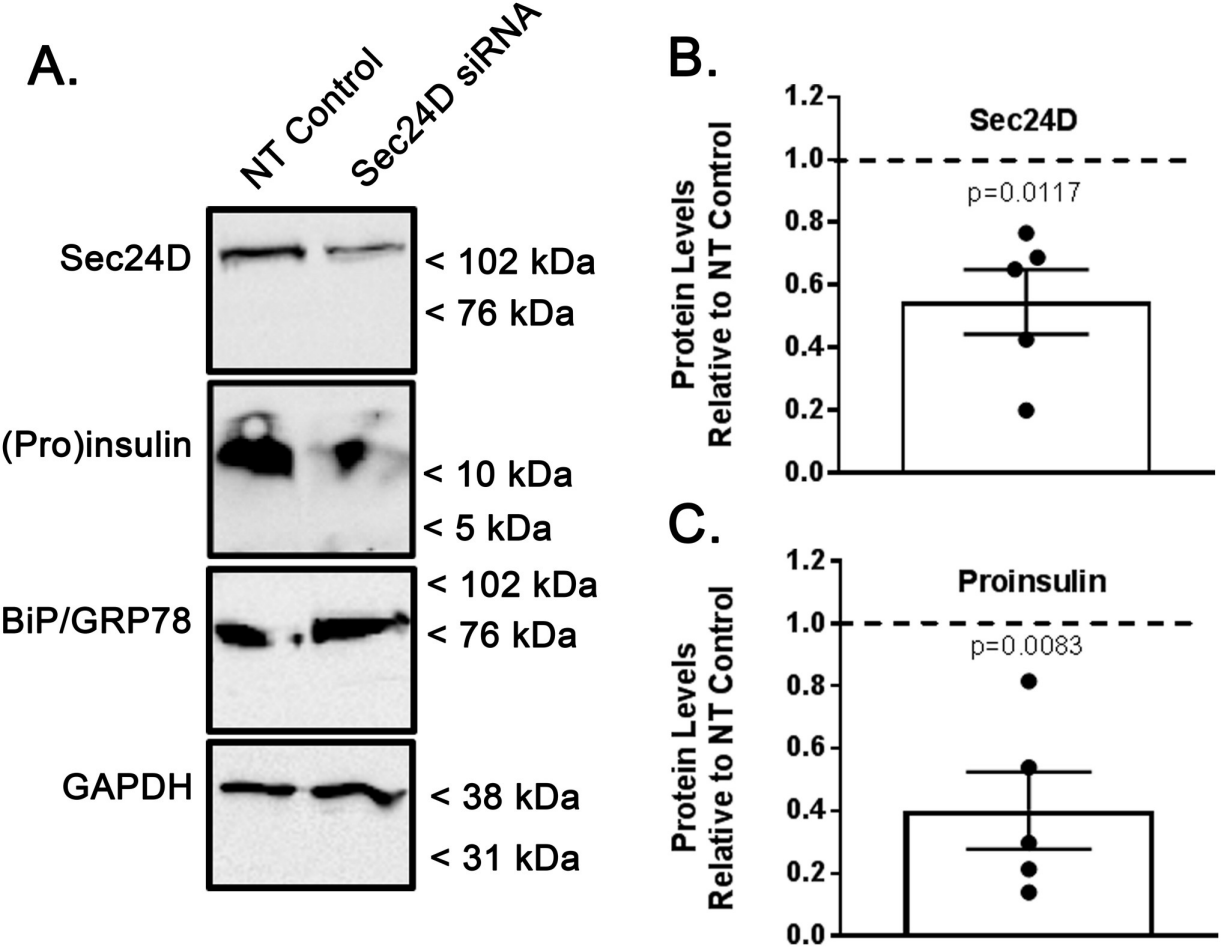
Sec24D regulates intracellular Proinsulin abundance. Min-6 cells were transiently transfected with non-targeted (control) or Sec24D-targeted siRNA. (A) Whole cell lysates were resolved by SDS-PAGE, followed by analysis by immunoblot. These data are representative of n = 5 independent experiments. (B) Densitometric quantification of the relative abundance of Sec24D in transiently transfected Min-6 cells indicate a decrease in Sec24D levels of ~50% when transfected with Sec24D-targeted siRNA vs. controls (n = 5, p = 0.0117). (C) Densitometric quantification of the relative abundance of Proinsulin indicate a corresponding decrease in whole cell lysate levels of ~60% when transfected with Sec24D-targeted siRNA vs. controls (n = 5, p = 0.0083).

To further assess the role of Sec24D in Proinsulin processing, Min-6 cells were transiently transfected with non-targeting (NT, control) or Sec24D-specific siRNA, and abundance of Sec24D and Proinsulin were subsequently analyzed by immunoblot (Fig 2A). Depletion of Sec24D was consistent across multiple experiments with an average of an ~50% decrease in whole cell lysate (Fig 2B, densitometry of n = 5 independent experiments, p = 0.0117 vs control). With Sec24D depletion, there was an overall decrease in total Proinsulin in the lysate (Fig 2A), with this depletion of Sec24D resulting in a corresponding ~60% decrease in whole cell Proinsulin abundance when quantified by densitometry (Fig 2C, densitometry of n = 5 independent experiments, p = 0.0083 vs control). ER stress was monitored by BiP/Grp78 levels. Densitometric quantification of BiP/Grp78 demonstrate that its relative abundance did not change significantly with Sec24D depletion as compared to controls (relative BiP/Grp78 abundance with Sec24D depletion 1.04 vs control, range 0.93–1.13, n = 5, p = 0.44), suggesting that ER stress was limited during these experiments. As most β-cell Proinsulin concentrates in the Golgi [33], it is possible that the observed decrease in intracellular Proinsulin content with Sec24D depletion indicates inhibited trafficking of Proinsulin from ER to Golgi, and a subsequent decrease in total capacity of cells to contain Proinsulin. Regardless, these observations support the hypothesis that Sec24D is a critical component in the biosynthesis of Insulin.

### Proinsulin, Sec24D, and ERp29 interact in Min-6 cells

**Fig 3:**
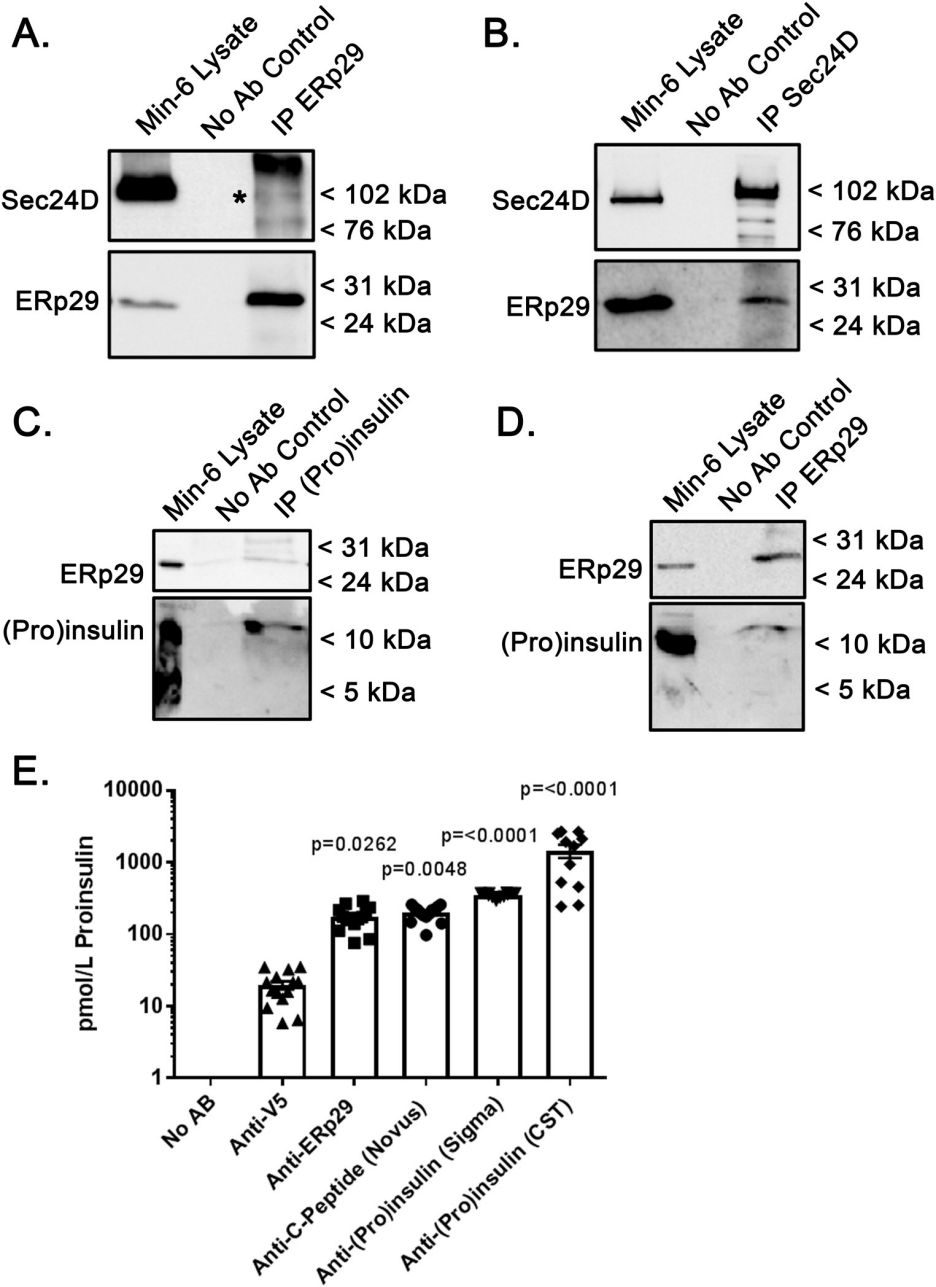
ERp29 interacts with Sec24D and Proinsulin. Min-6 cells were lysed under non-denaturing conditions, and 500 μg of total lysate protein was subject to immunoprecipitation with either anti-ERp29 (A, D), anti-Sec24D (B), or anti-(Pro)insulin (C). Precipitated proteins were resolved by SDS-PAGE and immunoblots were probed for Sec24D, ERp29, or (Pro)insulin as indicated. The Min-6 lysate lanes were loaded with 50 μg (10%) of the input lysate. These data are representative of n = 4 independent experiments. The co-precipitated Sec24D band is denoted with asterisk in panel (A). (E) Fifty μg of Min-6 whole cell lysate prepared under non-denaturing conditions was subject to immunoprecipitation under the following conditions: No antibody control (n = 12), anti-V5 (non-specific control, n = 11), anti-ERp29 (n = 14), anti-C-peptide from Novus (n = 14), anti-(Pro)Insulin from Sigma (n = 12), and anti-(Pro)Insulin from Cell Signaling Technologies (CST, n = 11). Precipitated Proinsulin was quantified by a rat/mouse Proinsulin ELISA from Mercodia as specified in the materials and methods. Anti-V5 control vs anti-ERp29, p = 0.0262; anti-V5 control vs anti-C-peptide, p = 0.0048; anti-V5 control vs Sigma antibody, p = <0.0001; anti-V5 control vs CST antibody, p = <0.0001.

Our previous work also demonstrated that ERp29 plays a critical role in directing the biosynthesis of the epithelial sodium channel, ENaC, likely in concert with Sec24D and via COP II-mediated ER→Golgi transport [20, 21]. Therefore, we tested whether this ERp29/Sec24D interaction was also present and perhaps relevant in Min-6 insulinoma cells using a co-immunoprecipitation approach. Min-6 whole cell lysates were subject to immunoprecipitation with anti-ERp29. Immunoblots of precipitated proteins demonstrated that both ERp29 and Sec24D precipitate with anti-ERp29, consistent with an association between ERp29 and Sec24D in pancreatic β-cells (Fig 3A, Sec24D denoted with asterisk). When the reverse experiment was performed with anti-Sec24D, both ERp29 and Sec24D precipitated with anit-Sec24D but did not precipitate in an antibody-free control experiment, further demonstrating an interaction between these two proteins in β-cells (Fig 3B). These data thus suggest that the interaction of ERp29 and Sec24D may be a more robustly conserved component of the COPII trafficking pathway, and is not an epithelial cell-specific phenomenon.

Due to the aforementioned putative ERp29 binding site on Proinsulin, as well as ERp29’s association with Sec24D, we hypothesized that ERp29 may interact in complex with Sec24D and Proinsulin. We therefore investigated this hypothesized interaction between Proinsulin and ERp29, using co-immunoprecipitation experiments in Min-6 cells. When immunoprecipitation was performed with anti-(Pro)Insulin, ERp29 co-precipitation was detected by immunoblot, as compared to a no-antibody control (Fig 3C). Similarly, immunoprecipitation with anti-ERp29 co-precipitated Proinsulin, while a no-antibody control did not (Fig 3D). In both immunoprecipitation experiments, antibodies appear to selectively precipitate Proinsulin, rather than mature Insulin, suggesting an interaction specifically between ERp29 and immature Proinsulin either directly or in complex.

The co-precipitation of Proinsulin with ERp29 was further quantified using ELISA after immunoprecipitation with anti-ERp29, anti-C-peptide, Sigma anti-(Pro)Insulin, and CST (Cell Signaling Technologies) anti-(Pro)Insulin. All resulted in >10 fold greater precipitation of proinsulin than immunoprecipitation with anti-V5 as a non-specific control (Fig 3E, anti-V5 control vs anti-ERp29, p = 0.0262; anti-V5 control vs anti-C-peptide, p = 0.0048; anti-V5 control vs Sigma, p = <0.0001; anti-V5 control vs CST, p = <0.0001). These data demonstrate that anti-ERp29 precipitates a comparable level of proinsulin to anti-C-peptide and two different (Pro)Insulin antibodies; only the CST (Pro)Insulin antibody precipitated significantly more Proinsulin than did anti-ERp29. Analogous experiments were performed in Ins-1 rat insulinoma cells, to confirm that the observed interactions were not species-specific, and revealed that ERp29 co-precipitates with (Pro)Insulin (S3A Fig). Similarly, ELISA quantification of co-precipitated Proinsulin in the reciprocal experiment where the immunoprecipitation was performed with anti-ERp29 revealed significant Proinsulin co-precipitation as compared to both anti-V5 and no antibody controls (S3B, anti-V5 Control vs anti-ERp29 p = <0.0001). Taken together, these data suggest that ERp29 interacts with Proinsulin, either directly or as part of a larger complex that likely includes the COP II cargo-recruiting component, Sec24D.

### Depletion of ERp29 decreases cellular Proinsulin

**Fig 4:**
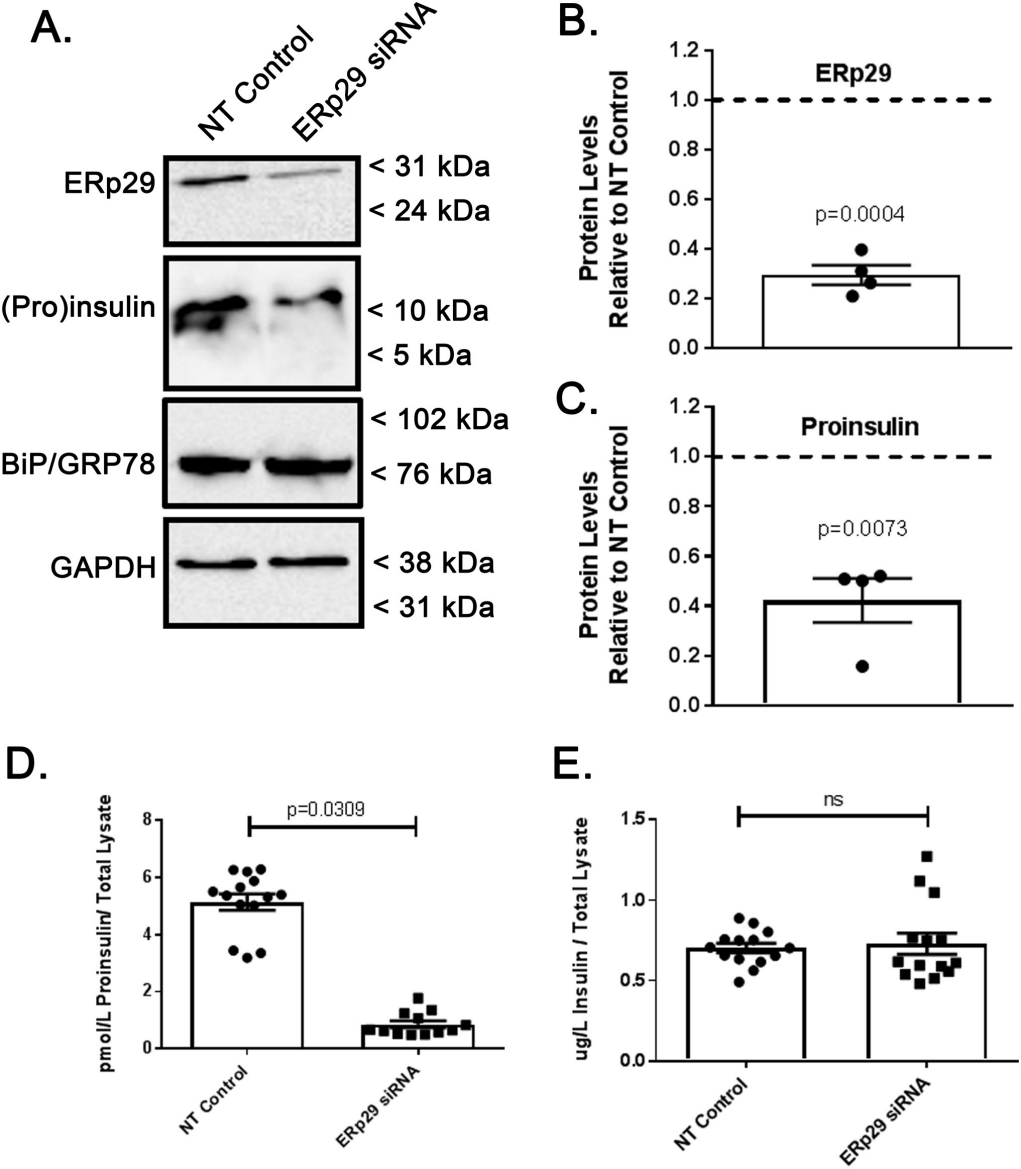
Depletion of ERp29 decreases cellular Proinsulin. Min-6 cells were transfected with ERp29-targeting siRNA or with a non-targeting control siRNA. (A) The resulting depletion of ERp29 and its effect on Proinsulin abundance were then assayed by immunoblot. GAPDH was used as a loading control, and BiP was used to monitor ER stress. These data are representative of n = 4 independent experiments. (B) Densitometric quantification of the relative abundance of ERp29 in transiently transfected Min-6 cells indicate a decrease in ERp29 levels of ~70% when transfected with ERp29-targeting siRNA vs. controls (n = 4, p = 0.004). (C) Densitometric quantification of the relative abundance of Proinsulin in transiently transfected Min-6 cells indicate a corresponding decrease in whole cell lysate levels of ~60% when transfected with ERp29-targeting siRNA vs. controls (n = 4, p = 0.0073). (D, E) Transiently transfected Min-6 cells were lysed and analyzed by ELISA. Proinsulin levels decreased when transfected with ERp29-targeting siRNA as compared to controls (D, p = 0.0309), but Insulin content did not significantly change (E, p = ns).

In order to further elucidate the role of ERp29 in Insulin biosynthesis, we tested whether altering the levels of intracellular ERp29 would affect the cellular abundance of Proinsulin. Min-6 cells were transiently transfected with siRNA targeting ERp29. As demonstrated by immunoblot (Fig 4A) and quantified by densitometry (Fig 4B), transfection with ERp29 siRNA resulted in ~70% decreased ERp29 abundance as compared to controls (Densitometry of n = 4 independent experiments, p = 0.0004 vs control). We also observed a corresponding ~60% decrease in cellular Proinsulin abundance when ERp29 was depleted (Fig 4A, representative immunoblot; Fig 4C, densitometry of n = 4 independent experiments, p = 0.0073 vs control). ER stress was again monitored by BiP/Grp78 levels, and densitometry revealed that the relative abundance of BiP/Grp78 did not change significantly with ERp29 depletion as compared to controls (relative BiP/Grp78 abundance with ERp29 depletion 1.07 vs control, range 0.93–1.12, n = 4, p = 0.37) suggesting that there was not significant ER stress during these experiments.

Intracellular Proinsulin (Fig 4D) and Insulin (Fig 4E) content in these transfected cell lysates were also assayed by ELISA in these experiments. These data confirm a significant decrease in cellular Proinsulin when ERp29 is depleted relative to non-targeting controls (Fig 4D; p = 0.0309). Interestingly, we did not observe a significant change in the total cellular Insulin content relative to non-targeting controls (Fig 4E; p = ns); this may reflect that cellular Insulin is present at much higher levels than Proinsulin and/or that most of the cellular Insulin has matured and is present prior to and throughout the course of this experiment. It is also possible that unaltered Insulin abundance with Sec24D depletion is indicative of poor or inconsistent transfection efficiency; however, other groups using similar methods to ours achieved consistent siRNA transfection efficiencies of 80–90% in Min-6 cells [34], and we observed consistent decreases in protein content of ~70%, so this explanation seems less likely. Nevertheless, these data together further support the hypothesis that ERp29 regulates intracellular Proinsulin levels and therefore, may play a role in Insulin maturation and biosynthesis.

### Overexpression of WT ERp29 increases cellular Proinsulin

**Fig 5:**
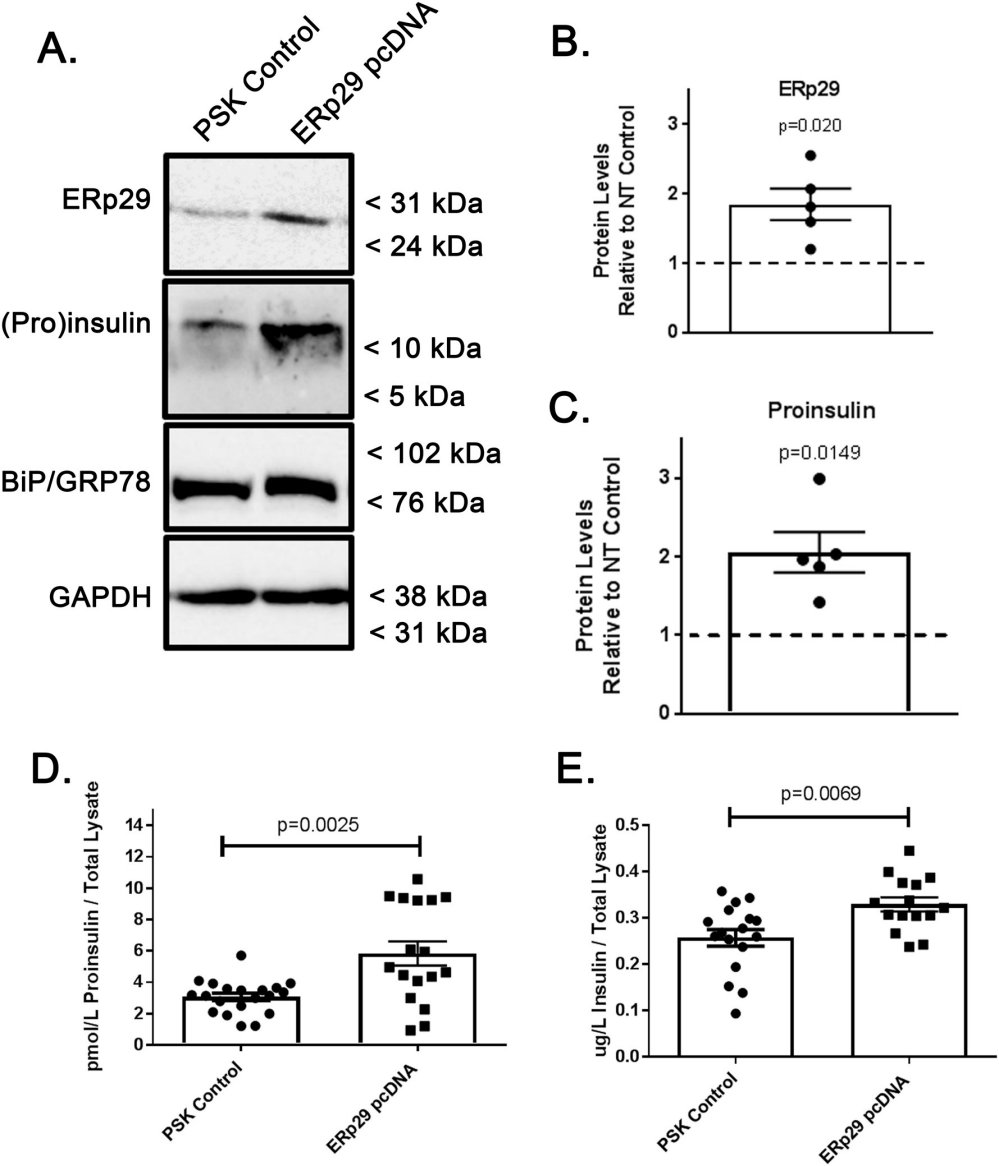
Overexpression of ERp29 increases cellular Proinsulin and Insulin. Min-6 cells were transiently transfected with a pcDNA4 plasmid expressing WT ERp29 or with a non-expressing PSK control plasmid, and whole cell lysates were prepared. (A) ERp29 and Proinsulin abundance was assayed in whole cell lysates by immunoblot. Immunoreactivity of GAPDH was used as a loading control, and immunoreactivity of BiP was used to monitor for ER stress. These data are representative of n = 5 independent experiments. Densitometry of ERp29 (B, p = 0.020) and Proinsulin (C, p = 0.0149) in immunoblots. (D, E) Lysates of transfected control or WT ERp29 transfected Min-6 cells were analyzed by ELISA, and data demonstrate an increase in both cellular Proinsulin (D, p = 0.0025) and Insulin (E, p = 0.0069) when compared to controls.

To further test this suggested role of ERp29 in regulating cellular Proinsulin levels and elucidate its potential role in Insulin biosynthesis, Min-6 cells were transiently transfected with plasmids containing wild type human ERp29 or a PSK empty vector control. Subsequently, whole cell ERp29 and Proinsulin protein content were analyzed by immunoblot and quantified with densitometry (Fig 5A, 5B and 5C). Densitometry measurements confirm an ~1.7-fold increase in cellular ERp29 levels with ERp29 WT pcDNA transfection when compared to controls (Fig 5B, densitometry of n = 5 independent experiments, p = 0.020 vs control). Proinsulin blot quantification with densitometry revealed a corresponding ~2-fold increase in cellular Proinsulin content with ERp29 WT overexpression relative to control transfections (Fig 5C, n = 5, p = 0.0149 vs control). Again, the relative abundance of BiP, a marker of ER stress, was consistent throughout these experiments, suggesting a minimal contribution of ER stress to our results (relative BiP/Grp78 abundance with ERp29 overexpression 0.98 vs control, range 0.87–1.04, n = 5, p = 0.44, representative immunoblot in Fig 5A). As before, we used ELISA to further quantify and confirm this observed increase in cellular Proinsulin content as a result of WT ERp29 overexpression (Fig 5D, p = 0.0025). Interestingly, Insulin ELISA data also revealed a small but significant increase in cellular Insulin content in lysates of cells transiently transfected with WT ERp29 plasmid as compared to controls (Fig 5E; p = 0.0069). These data further suggest a role for ERp29 in regulating cellular Proinsulin levels, perhaps via stabilization, and importantly, are the first data to suggest a role for ERp29 in promoting Insulin maturation during biosynthesis.

### Overexpression of mutant ERp29 C157S alters Proinsulin processing

**Fig 6:**
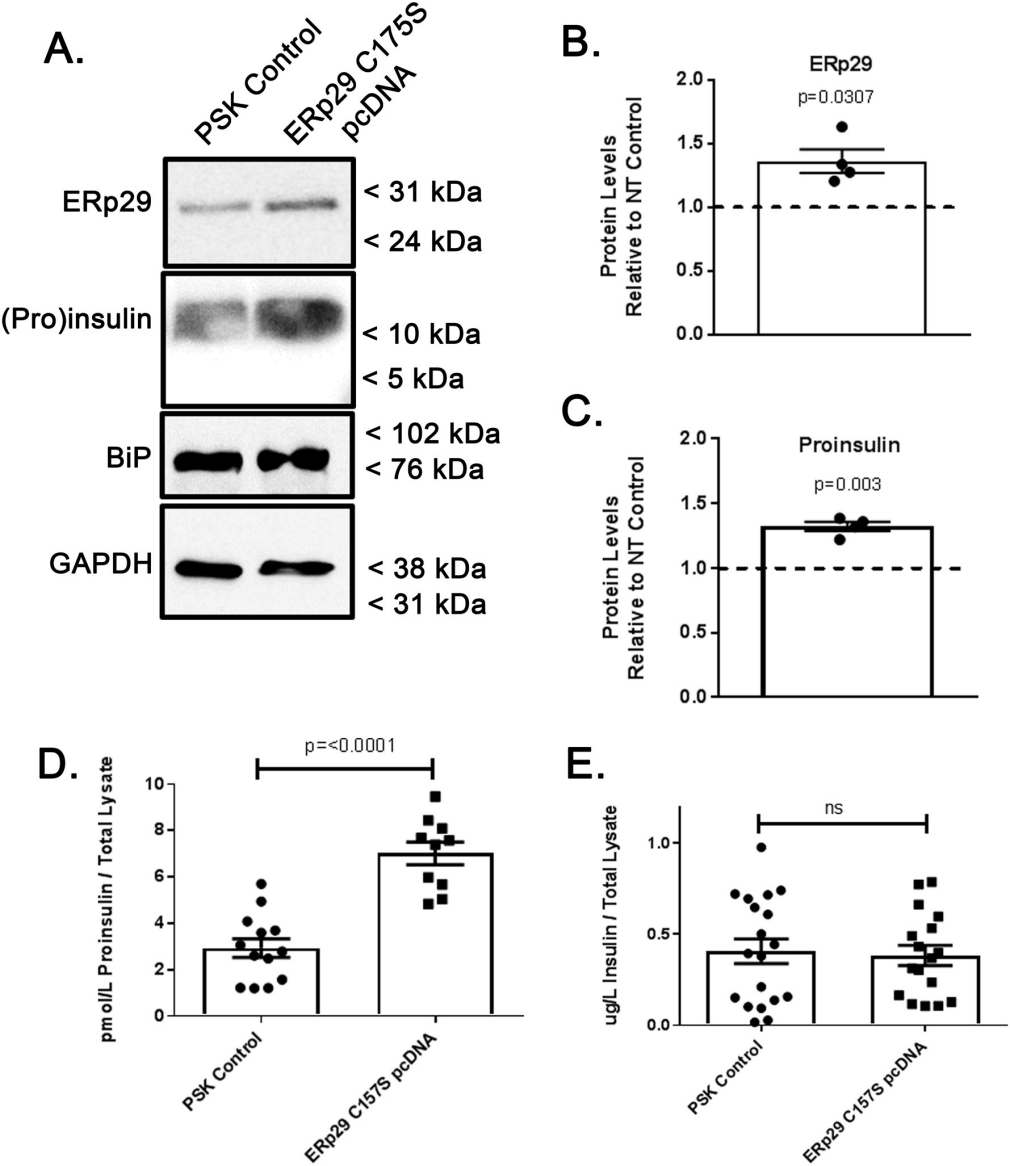
Overexpression of ERp29 C157S increases cellular Proinsulin, but not cellular Insulin. Min-6 cells were transiently transfected with ERp29 C157S pcDNA4 plasmid or mock transfected with non-expressing PSK plasmid as a control. Whole cell lysates were prepared and (A) ERp29 and Proinsulin protein abundance was assayed in whole cell lysates with immunoblot. Immunoreactivity of GAPDH was used as a loading control, and immunoreactivity of BiP was used to monitor for ER stress. These data are representative of n = 4 independent experiments. (B) Densitometry of ERp29 abundance as assessed by immunoblot reveals an ~40% increase in protein levels in ERp29 C157S transfected lysates, relative to controls (p = 0.0307). (C) Densitometry of Proinsulin abundance in immunoblots demonstrates a corresponding increase of ~40% with ERp29 C157S overexpression (p = 0.003). (D, E) Lysates of transfected control or ERp29 C157S transfected Min-6 cells were analyzed by ELISA for cellular content of Proinsulin (D, p = <0.0001) and Insulin (E, p = ns).

ERp29 has a single, evolutionarily conserved Cysteine (C157) that is not implicated in client interaction or in catalyzing client disulfide formation or exchange [27]. However, our group’s previous data [21] demonstrate that expression of a mutant ERp29 where the single cysteine is converted to serine (C157S) inhibits ERp29 function with regard to ENaC biosynthesis in a dominant-negative manner. We therefore tested whether expression of ERp29 C157S would result in a similar effect with respect to Insulin biosynthesis. Min-6 cells were transfected with plasmids encoding the mutant ERp29 C157S, and subsequently, the protein content of the whole cell lysates were analyzed by both immunoblot (Fig 6A) and ELISA (Fig 6D and 6E). An approximately 40% increase in ERp29 levels, as quantified with densitometry, was confirmed in ERp29 C157S-transfected cells as compared to controls, presumably representing effective expression of plasmid ERp29 C157S (Fig 6B, densitometry of n = 4 independent experiments, p = 0.0307 vs control), and this increase in expression resulted in a corresponding, ~40% increase in cellular Proinsulin as compared to transfection controls (Fig 6C, n = 4, p = 0.003 vs control). Again, there was no significant ER stress in these experiments as assessed by BiP/Grp78 levels (relative BiP/Grp78 abundance with ERp29 C157S overexpression 1.04 vs control, range 0.94–1.07, n = 5, p = 0.44).

This increase in Proinsulin abundance upon transfection of ERp29 C157S was confirmed by ELISA (Fig 6D, p = <0.0001) and recapitulated what was observed with overexpression of WT ERp29 (Fig 5D). However, ERp29 C157S overexpression did not result in a corresponding increase in mature Insulin (Fig 6E, p = ns), as was observed with ERp29 WT overexpression (Fig 5E, p = <0.0001); instead, no significant increase in Insulin content was observed upon expression of ERp29 C157S, when measured with ELISA (Fig 6E). These data suggest that while both WT ERp29 and ERp29 C157S might stabilize and therefore increase cellular Proinsulin, only (over)expression of WT ERp29 increases cellular Insulin content. Thus, only WT ERp29 appears to promote Insulin biosynthesis. It is possible that mutant ERp29 C157S retains its ability to interact in some manner with Proinsulin, but cannot promote its maturation into Insulin, as WT ERp29 overexpression does. In fact, ERp29 C157S likely retains the ability to interact with its client proteins, as the C157 motif is outside of its client-binding site [21]. These data parallel our previous work, which demonstrated that ERp29 C157S expression (as compared to ERp29 WT overexpression) does not promote ENaC biosynthesis by directing it for cleavage in the Golgi. Taken together, these data further support the hypothesis that ERp29 plays a role in promoting Insulin biosynthesis and maturation, perhaps through stabilization of immature Proinsulin and/or through directing Proinsulin for cleavage in the trans-Golgi.

## Discussion

The environment within the ER and ER stress play an important role in protein biosynthesis, processing, and secretion. ER stress is hypothesized to play a critical role in modulating Insulin biosynthesis, β-cell failure, and apoptosis in T2D and CFRD, but the underlying mechanisms that facilitate “healthy” ER function in β-cells have not been fully elucidated. Here we demonstrate that ERp29, a novel ER chaperone [35], regulates the abundance of Proinsulin and its association with COP II machinery in mammalian models of pancreatic β-cells.

**Fig 7:**
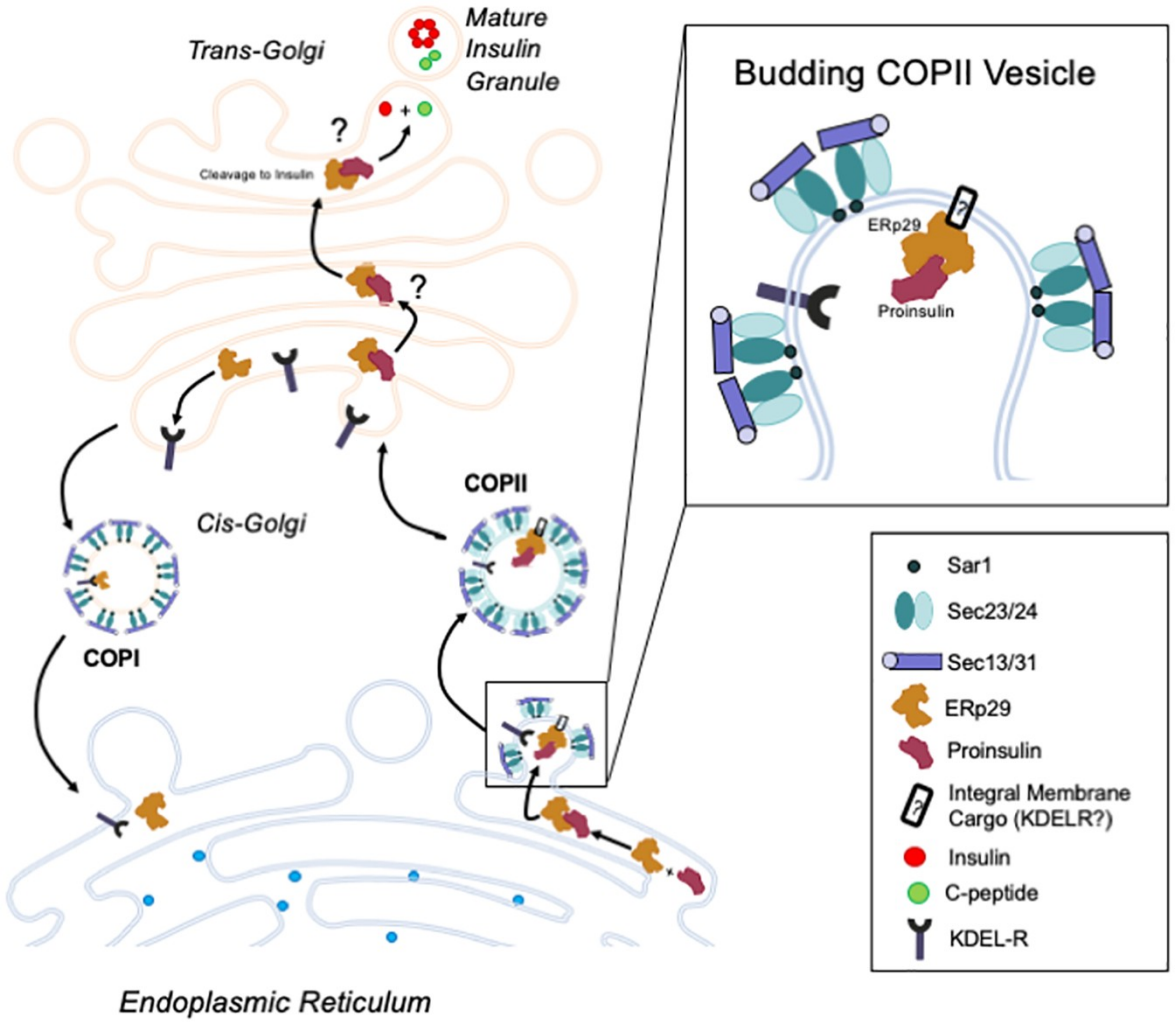
Hypothesized role for ERp29 in Insulin synthesis. Proinsulin (Red) is transported from the ER to the cis-Golgi via COP II vesicles; however, it is currently unclear how Proinsulin is recruited for inclusion in COP II vesicles. The Sec23/24 heterodimer is an essential component of the COP II vesicle pre-budding complex, which also includes Sar1 and the Sec13/31 heterodimer. Our data demonstrate that the Sec24 isoform, Sec24D, interacts with both ERp29 and Proinsulin in pancreatic β-cells, likely as part of a complex. We hypothesize that ERp29 interacts directly with Proinsulin at its putative ERp29 binding domain (^24^F-F-Y^26^ in Proinsulin) and promotes inclusion of the Proinsulin/ERp29 complex in COP II vesicles. In this model, ERp29 also associates with a currently unknown integral membrane protein that intermediates the interaction of Proinsulin/ERp29 with Sec24D. One such integral membrane protein may be the KDEL receptor (KDEL-R), which cycles between the ER and cis-Golgi via COP I and II vesicles. Our group previously demonstrated that KDEL-R facilitates the transport of other COP II cargo, specifically the epithelial sodium channel, ENaC, from ER to Golgi, in concert with ERp29 and Sec24D. Future studies will test the hypothesis that KDEL-R intermediates the interaction of ERp29/Proinsulin with Sec24D. Our data also suggest that ERp29 may stabilize Proinsulin, perhaps as it transverses the Golgi cisternae to the trans-Golgi, where it is cleaved to mature Insulin and C-peptide and packaged into granules for secretion.

It is currently unclear how, in native conditions, a pool of Proinsulin is directed for processing and cleavage into Insulin and C-peptide, while another pool is directed for secretion in its uncleaved, non-functional form, perhaps bypassing COP II vesicle transport at the ER export stage. The importance of maintaining this sorting process is evidenced by the increased ratio of secreted Proinsulin to Insulin in pre-T2D and CF [10–12]. Previous work has demonstrated that the Sar1 GTPase, which initiates the pre-budding complex for COP II vesicle formation (as depicted in Fig 7) is necessary for proper biosynthesis of Proinsulin, and that inhibition of Sar1 precludes the cleavage of Proinsulin into mature Insulin and C-peptide [19]. Without Sar1 activity, COP II vesicle formation is presumably not initiated, so Proinsulin is not directed to the Golgi for further processing. Other COP II pre-complex components, including Sec24, are involved in recruiting both integral membrane cargo and membrane-associated proteins for export from the ER [17, 18]. Here, and consistent with these previous data, we demonstrate that Sec24D associates with Proinsulin, and we further demonstrate that Sec24D functions in concert with the ER-resident chaperone protein, ERp29, potentially to support Proinsulin maturation in Min-6 cells. Together our data suggest a role for ERp29 in directing Proinsulin for cleavage and maturation, perhaps via facilitation of Proinsulin’s inclusion into COP II vesicles and/or *via* stabilization of Proinsulin during biosynthesis, as depicted in Fig 7.

Given the suggested importance of COP II pre-complex formation in the processing and biosynthesis of Proinsulin, it seemed likely that Sec24D, one of the five key components of the pre-budding complex, would play a key role in Insulin biosynthesis. Our data support this notion, and demonstrate that Sec24D depletion resulted in decreased intracellular Proinsulin abundance, a result that parallels those previously observed with either inhibition or depletion of Sar1 [19]. Recent work suggests that Proinsulin predominantly localizes to the Golgi [33]. It is therefore possible that inhibited ER to Golgi transport, as a result of Sec24D depletion, precludes the accumulation of Proinsulin in the Golgi. This would likely result in increased Proinsulin abundance in the ER, which, in turn, could initiate ERAD-mediated degradation of Proinsulin. However, upregulated ERAD in the absence of misfolded protein would suggest the presence of ER stress (ERS), which was not indicated through observation of BiP/Grp78 abundance; although we cannot preclude the possibility that there may have been changes in expression of other stress-responsive genes. Alternatively, it is possible that inhibition of COP II-mediated ER exit, either through Sec24D depletion (Fig 2) or Sar1 inhibition [19], results in increased secretion of Proinsulin via an alternative pathway, perhaps the constitutive pathway, in which Proinsulin bypasses cleavage in the trans-Golgi and is secreted in its immature form. It is important to note that secretion via the constitutive pathway is not dependent on glucose stimulation or membrane depolarization [36]. It is therefore possible that immature Proinsulin that is not transported to the Golgi via COP II vesicle transport, may exit the ER through an alternative method that results in a higher rate of immature Proinsulin secretion and decreased intracellular Proinsulin content.

Interestingly, we did not observe a change in intracellular Insulin content with Sec24D depletion, measured by ELISA, as was seen with Sar1 inhibition. This incongruence could result from an incomplete blockage of COP II vesicle formation, either due to partial depletion of Sec24D via siRNA in our experiments, or due to differences in the functional necessity of Sec24 vs Sar1 for COP II vesicle formation. The Sec23/24 heteroduplex is functionally required to recruit the Sec13/30 heteroduplex, which serves to initiate membrane deformation and budding at the ER surface [17] (Fig 7). While Sar1 is required to initiate this process, it is possible that decreased Sec24 abundance has a less severe effect on pre-budding complex formation than complete inhibition of Sar1. Nevertheless, our data are consistent with Sec24D playing a role in Proinsulin biosynthesis that is likely similar to that of Sar1.

The mechanism by which Proinsulin is recognized or sorted for exit from the ER is currently unclear, and there is evidence to suggest that a COP II-independent ER exit pathway also exists [37, 38]. ERp29 is required for the proper biosynthesis of CFTR and corrects the aberrant trafficking of F508del [25]. Our group has also demonstrated that ERp29 promotes the cleavage and activation of ENaC during its biosynthesis [21], a pathway that involved cleavage of ENaC in the Golgi or later compartments [29, 39]. Although Insulin is neither an ion channel nor an integral membrane protein, when examined closely, there are key similarities between ENaC and Insulin biosynthesis that portend a role for ERp29 in Insulin biosynthesis. First, both Proinsulin and ENaC can be processed by furin-like convertases in the Golgi or later compartments into a more active from (Insulin and cleaved, higher P_o_ ENaC, respectively). Second, both proteins are transported from the ER to the Golgi by COP II machinery. Third, both Proinsulin and ENaC can bypass this processing in the Golgi to be secreted (as Proinsulin) or arrive at the apical membrane (as uncleaved, low P_o_ ENaC) in a less active form. Finally, elevated secreted Proinsulin/Insulin ratios are seen in both CF and pre-T2D, suggesting impaired Insulin processing, perhaps due to ER stress [10–12]. These similarities led us to test the hypothesis that ERp29 is a critical factor in promoting the conversion of Proinsulin to Insulin.

Here, we confirm that ERp29 interacts with COP II pre-complex component, Sec24D in models of pancreatic β-cells in co-precipitation experiments, (Fig 3) as was previously demonstrated in epithelial cell lines by our group [20]. Additionally, we demonstrate that ERp29 interacts with Proinsulin (Fig 3) in similar co-precipitation experiments, either directly or in complex with other proteins, further suggesting a potential role for ERp29 in Proinsulin sorting at the stage of COP II vesicle formation and ER exit. We also demonstrated that partial depletion of ERp29 results in decreased cellular Proinsulin without altering cellular Insulin content (Fig 4), while overexpression of WT ERp29 results in both increased cellular Proinsulin AND mature Insulin (Fig 5). We therefore suggest that WT ERp29 promotes Proinsulin processing and maturation, and may also stabilize intracellular Proinsulin prior to maturation. ERp29 has a single Cysteine (C157) that is not implicated in client interaction or catalyzing client disulfide formation/exchange [27] and is extrinsic to its client binding site [28]. Our group’s previous data [21] suggest that mutation of this Cys to Ser (C157S) inhibits ERp29 function with regards to ENaC biosynthesis, in what appears to be a dominant-negative manner. Therefore, we investigated the implications of this mutant in Insulin biosynthesis, and found that expression of ERp29 C157S, like overexpression of WT ERp29, resulted in increased intracellular Proinsulin. However, expression of ERp29 C157S did not result in increased cellular mature Insulin (Fig 6E), which contrasts the effect of WT ERp29 overexpression (Fig 5E). Taken together, these data suggest that ERp29 both stabilizes Proinsulin and promotes its cleavage into its mature form. Furthermore, ERp29’s C157 motif appears to be necessary to promote Proinsulin cleavage and maturation, but is perhaps unnecessary for ERp29’s role in stabilization of Proinsulin; this conclusion is supported by the notion that C157 is not involved in client binding. Of significance, Ins gene expression levels were not monitored during these experiments and it is possible that our results reflect changes in expression, as opposed to stabilization; however, ERp29 is a chaperone protein that does not appear to mechanistically act to increase transcription of its other client proteins, including CFTR [25].

In summary, we hypothesize that ERp29 works in concert with the COP II cargo-recruiting protein, Sec24D, to promote the inclusion of Proinsulin in COP II vesicles, and may also stabilize Proinsulin prior to cleavage into its mature form (Fig 7). We suggest that ERp29 interacts directly with Proinsulin in the ER via its putative ERp29 binding domain (^24^F-F-Y^26^) and therefore promotes the concentration of Proinsulin at the COP II vesicle budding sites on the ER membrane (Fig 7). In this case, a currently unknown integral membrane intermediate would be required to complete the complex between cytosolic Sec24D and luminal ERp29/Proinsulin, denoted with a question mark in Fig 7. Our group recently demonstrated that one such integral membrane protein, the KDEL receptor (KDEL-R) complexes with both ERp29 and Sec24D, and works in concert with these proteins to promote the maturation of ENaC [20]. Future studies will test the hypothesis that KDEL-R plays a similar role in forward trafficking of Proinsulin. Of additional interest, ERp29 prevents the premature hexamerization of Connexin43 hemichannels through stabilization of the monomeric subunits in the ER and “escorts” these monomers to the Golgi where normal hexamerization occurs [40]. By analogy, ERp29 may similarly prevent premature hexamerization of Proinsulin. Therefore, future studies should also test a potential role for ERp29 in regulating the dynamics and timing of Proinsulin hexamerization.

ERp29 is hypothesized to interact with either Y/F-X-Y/F or Y/F-Y/F motifs on client proteins, and Pre-Proinsulin has one such motif ^48^F-F-Y^50^ (^24^F-F-Y^26^ in Proinsulin). Interestingly, naturally occurring mutations of F48 result in defects in Insulin biosynthesis. F48C causes neonatal diabetes by preventing Insulin secretion in a dominant negative fashion [41], while F48S causes hyperinsulinemic diabetes characterized by reduced C-peptide/Insulin ratios suggesting reduced cleavage of circulating “Insulin,” and reduced Insulin and C-peptide secretion in response to glucose stimulation [42, 43]. It is important to note that the conclusions drawn from our results are limited, as we have not directly assessed human islets or β-cells. Nevertheless, as ERp29 and COP II-mediated ER exit is highly evolutionarily conserved [19], our conclusions are likely applicable across mammalian models. Future studies to test the hypothesis that these mutant Insulins have defective biosynthesis because they do not interact with ERp29 will give fundamental insight into our understanding of the regulation of Insulin biosynthesis.

## Supporting information

S1 Fig Standardization of ELISA for analysis. These experiments confirm the specificity of the Proinsulin and Insulin ELISA kits used in this work, and verified manufacturers’ specifications for these kits. (A) Analysis of Proinsulin Standards from Mercodia Rat/Mouse Proinsulin kit using Mercodia Insulin ELISA kits (n = 4). Cross reactivity of Proinsulin in the Insulin ELISA was observed and is consistent with manufacturer’s specifications (n = 4). (B) Analysis of Insulin Standards using Rat/Mouse Proinsulin ELISA from Mercodia. Error bars that are not visible are contained within the respective data point. Insulin was not detected by the Proinsulin ELISA kit, which again, is consistent with manufacturer’s specifications. (C, D) To further establish the specificity of the Mercodia Rat/Mouse Proinsulin and Insulin ELISA kits, recombinant, purified C-peptide, Proinsulin and Insulin were analyzed in decreasing concentrations in duplicate. (C) The Mercodia Rat/Mouse Proinsulin ELISA robustly detected human Proinsulin, and showed no cross reactivity for human C-peptide or human Proinsulin. (D) The Mercodia Rat/Mouse Insulin ELISA detected Human Proinsulin and Insulin, (at a much higher affinity) and showed no cross reactivity for human C-peptide. These data are consistent with the manufacturers reported cross reactivity specifications.
(TIF)

S2 Fig Proinsulin interacts with Sec24D in Ins-1 Cells. Ins-1 rat insulinoma cells were lysed under non-denaturing conditions. (A) 500 μg of whole cell lysate protein was subject to immunoprecipitation with an anti-Insulin/Proinsulin. The precipitated proteins were resolved by SDS-PAGE and immunoblots were probed for Sec24D or Proinsulin. 50 μg of whole cell lysate protein (10% of input) was loaded in the Ins-1 lysate lane. (B) 50 μg of whole cell lysate protein was subject to immunoprecipitation with a no antibody control (n = 11), anti-V5 (non-specific antibody control, n = 11), or anti-Sec24D antibody (n = 8). Proinsulin in the precipitated proteins was quantified by ELISA. Anti-V5 control vs anti-Sec24D p = <0.0001.
(TIF)

S3 Fig Proinsulin interacts with Sec24D and ERp29 in Ins-1 Cells. Ins-1 cells were lysed under non-denaturing conditions. (A) 500 μg of whole cell lysate protein was subject to immunoprecipitation with an anti-C-peptide (anti-(Pro)Insulin). The precipitated proteins were resolved by SDS-PAGE and immunoblots were probed for Sec24D. 50 μg of whole cell lysate protein (10% of input) was loaded in the Ins-1 lysate lane. (B) 50 μg of lysate was subject to immunoprecipitation with a no antibody control (n = 9), anit-V5 (non-specific antibody control, n = 11), or anti-ERp29 (n = 10). Proinsulin content in the precipitated protein was determined by ELISA. Anti-V5 Control vs anti-ERp29, p = <0.0001.
(TIF)

S1 Raw images Raw immunoblot images were detected with chemiluminescent Substrate from Thermo (34080) and imaged on a BioRad ChemiDocTouch imaging system. (PDF)

S1 File Compiled data. Excel sheet with numerical data from densitometry and ELISA broken down by figure.
(XLSX)
