# Correction: ERp29 as a regulator of Insulin biosynthesis

**DOI:** 10.1371/journal.pone.0238264

**Published:** 2020-08-21

**Authors:** Jeffrey Viviano, Margaret Brecker, Christine Ferrara-Cook, Laurence Suaud, Ronald C. Rubenstein

## Notice of Republication

An incorrect version of Fig 7 was published in error. This article was republished on August 11, 2020 to correct for this error. Please download this article again to view the correct version.
