# Supplementary figures and images for: ERp29 as a regulator of Insulin biosynthesis
Source: PLoS One. 2020 May 20;15(5):e0233502. doi: 10.1371/journal.pone.0233502 (PMC7239452; doi:10.1371/journal.pone.0233502)

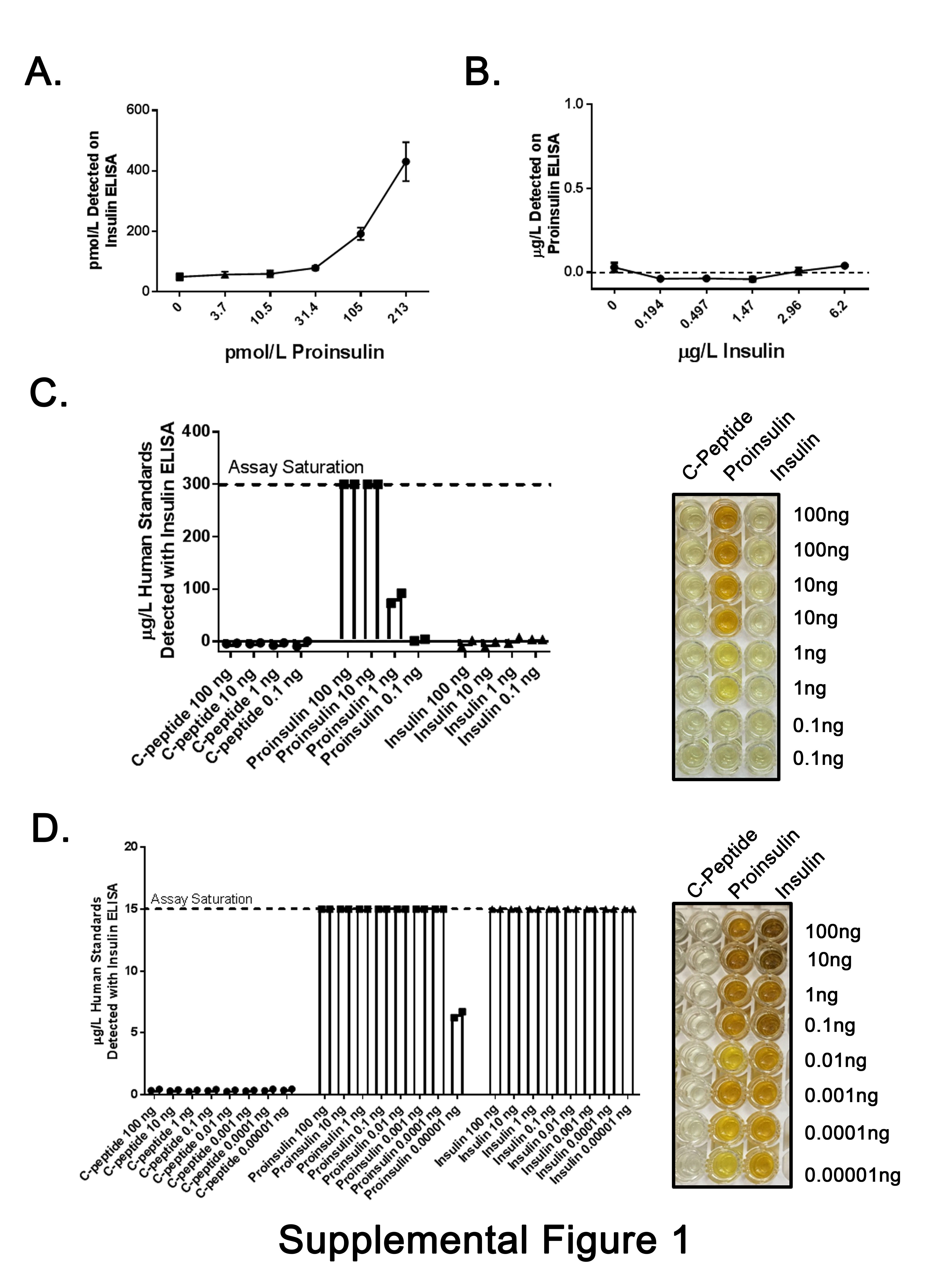

Supplement: S1 Fig — These experiments confirm the specificity of the Proinsulin and Insulin ELISA kits used in this work, and verified manufacturers’ specifications for these kits. (A) Analysis of Proinsulin Standards from Mercodia Rat/Mouse Proinsulin kit using Mercodia Insulin ELISA kits (n = 4). Cross reactivity of Proinsulin in the Insulin ELISA was observed and is consistent with manufacturer’s specifications (n = 4). (B) Analysis of Insulin Standards using Rat/Mouse Proinsulin ELISA from Mercodia. Error bars that are not visible are contained within the respective data point. Insulin was not detected by the Proinsulin ELISA kit, which again, is consistent with manufacturer’s specifications. (C, D) To further establish the specificity of the Mercodia Rat/Mouse Proinsulin and Insulin ELISA kits, recombinant, purified C-peptide, Proinsulin and Insulin were analyzed in decreasing concentrations in duplicate. (C) The Mercodia Rat/Mouse Proinsulin ELISA robustly detected human Proinsulin, and showed no cross reactivity for human C-peptide or human Proinsulin. (D) The Mercodia Rat/Mouse Insulin ELISA detected Human Proinsulin and Insulin, (at a much higher affinity) and showed no cross reactivity for human C-peptide. These data are consistent with the manufacturers reported cross reactivity specifications. (TIF) [file pone.0233502.s001.tif]

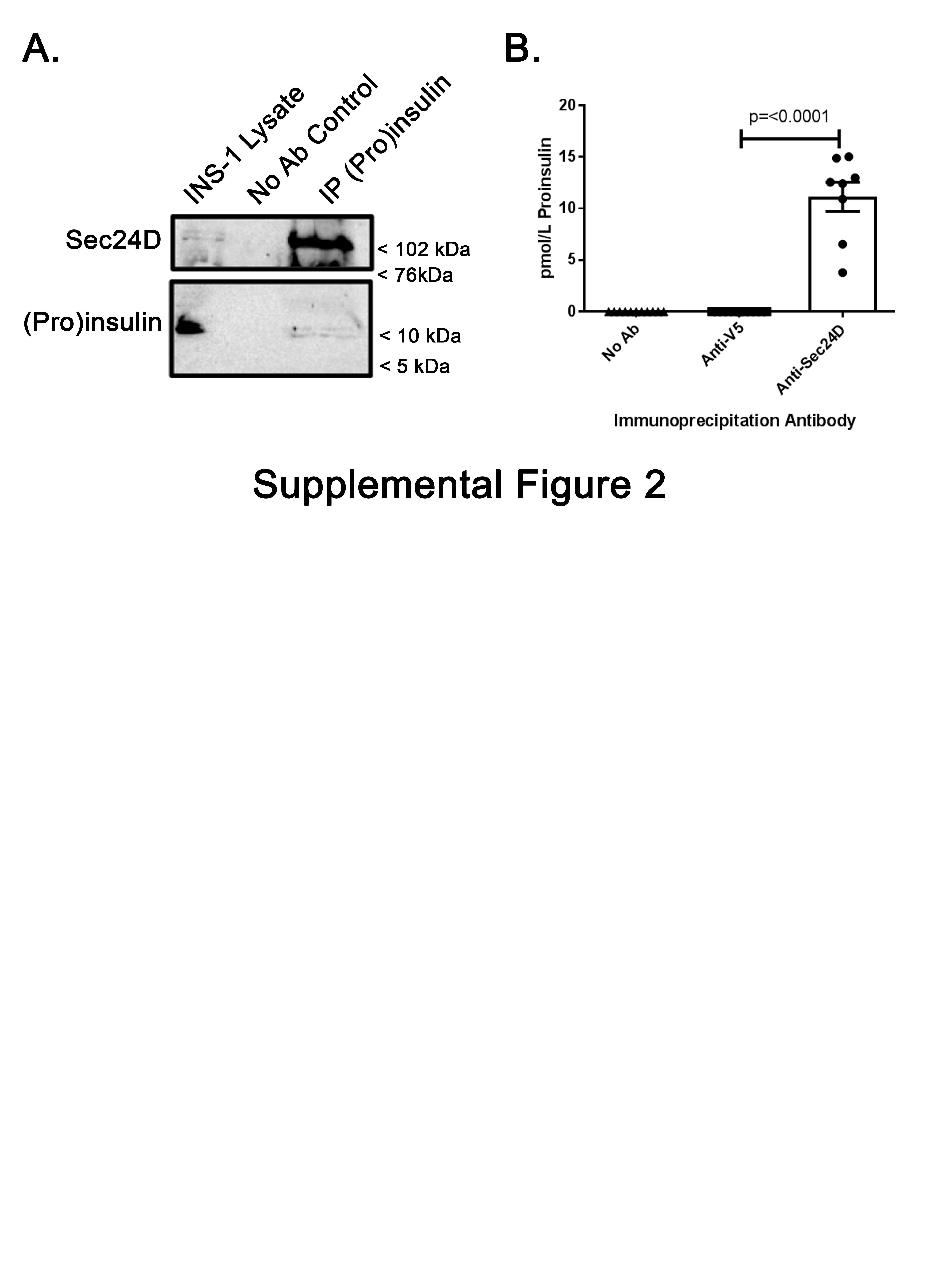

Supplement: S2 Fig — Ins-1 rat insulinoma cells were lysed under non-denaturing conditions. (A) 500 μg of whole cell lysate protein was subject to immunoprecipitation with an anti-Insulin/Proinsulin. The precipitated proteins were resolved by SDS-PAGE and immunoblots were probed for Sec24D or Proinsulin. 50 μg of whole cell lysate protein (10% of input) was loaded in the Ins-1 lysate lane. (B) 50 μg of whole cell lysate protein was subject to immunoprecipitation with a no antibody control (n = 11), anti-V5 (non-specific antibody control, n = 11), or anti-Sec24D antibody (n = 8). Proinsulin in the precipitated proteins was quantified by ELISA. Anti-V5 control vs anti-Sec24D p = <0.0001. (TIF) [file pone.0233502.s002.tif]

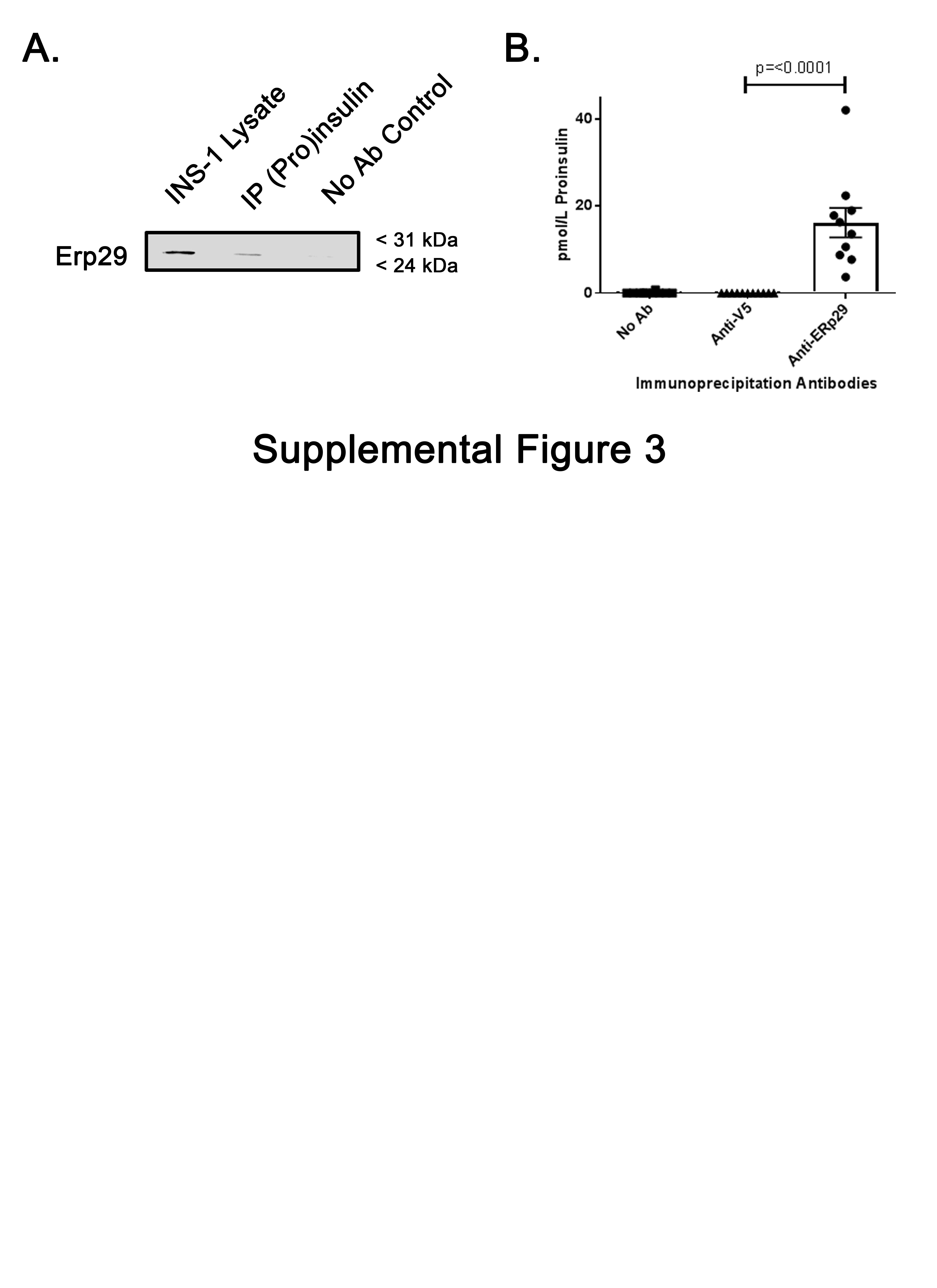

Supplement: S3 Fig — Ins-1 cells were lysed under non-denaturing conditions. (A) 500 μg of whole cell lysate protein was subject to immunoprecipitation with an anti-C-peptide (anti-(Pro)Insulin). The precipitated proteins were resolved by SDS-PAGE and immunoblots were probed for Sec24D. 50 μg of whole cell lysate protein (10% of input) was loaded in the Ins-1 lysate lane. (B) 50 μg of lysate was subject to immunoprecipitation with a no antibody control (n = 9), anit-V5 (non-specific antibody control, n = 11), or anti-ERp29 (n = 10). Proinsulin content in the precipitated protein was determined by ELISA. Anti-V5 Control vs anti-ERp29, p = <0.0001. (TIF) [file pone.0233502.s003.tif]
