# Supplementary material for: ERp29 as a regulator of Insulin biosynthesis
Source: PLoS One. 2020 May 20;15(5):e0233502. doi: 10.1371/journal.pone.0233502 (PMC7239452; doi:10.1371/journal.pone.0233502)

Figure 1A Original Blots

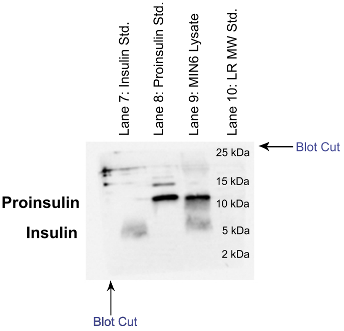

Figure 1B Original Blots

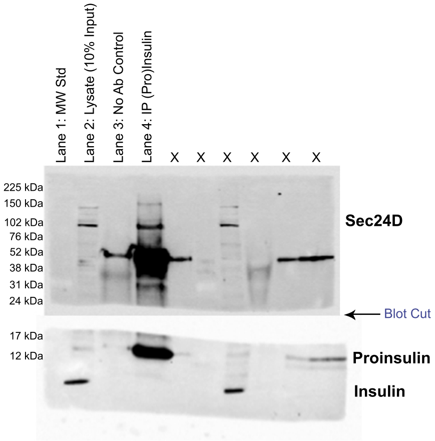

Figure 2A Original Blots

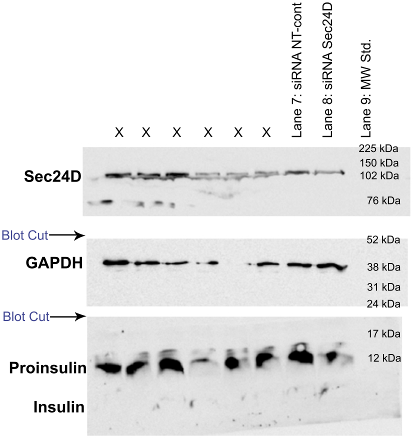

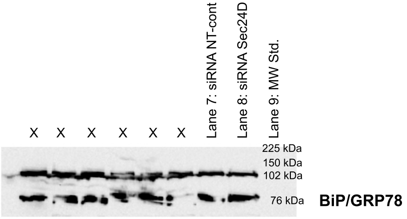

Same Blot probed BiP

Figure 3A Original Blots

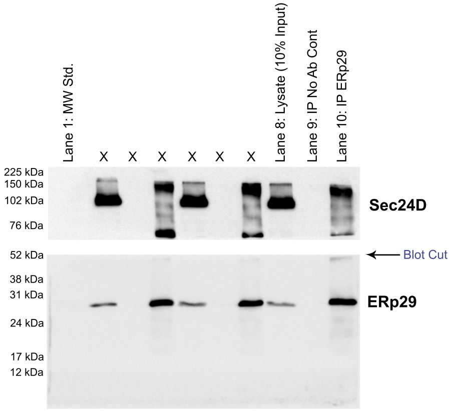

Figure 3B Original Blots

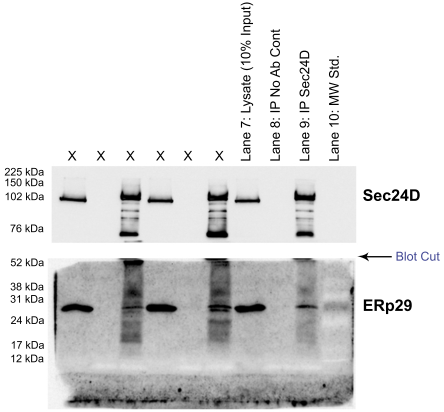

Figure 3C Original Blots

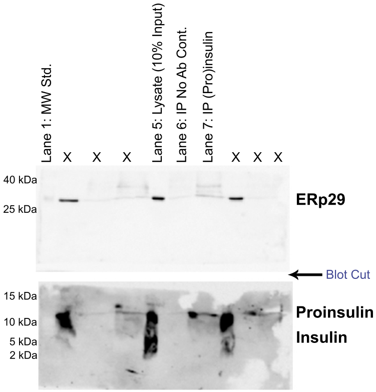

Figure 3D Original Blots

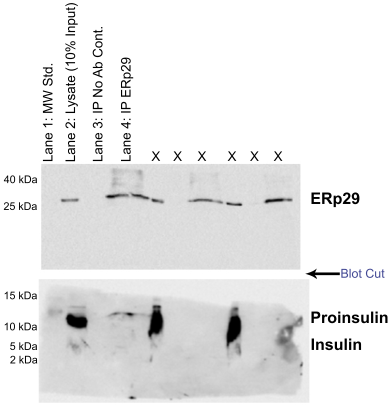

Figure 4A Original Blots

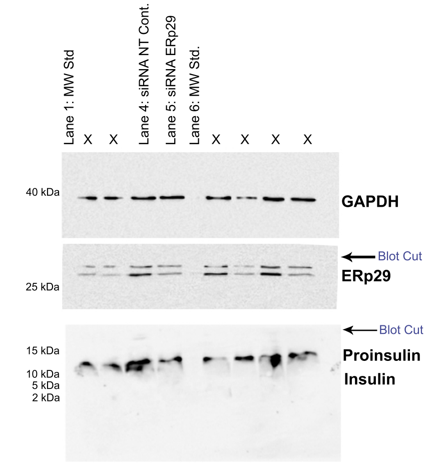

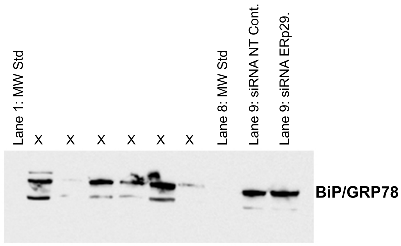

Separate Blot

Figure 5A Original Blots

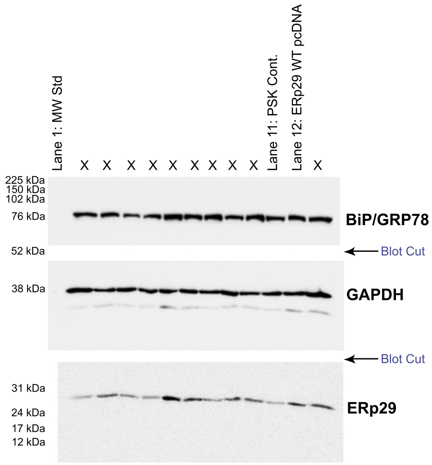

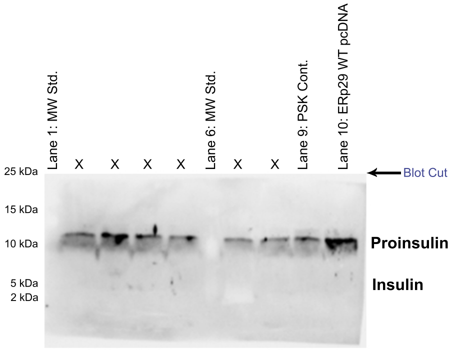

Separate Blot

Figure 6A Original Blots

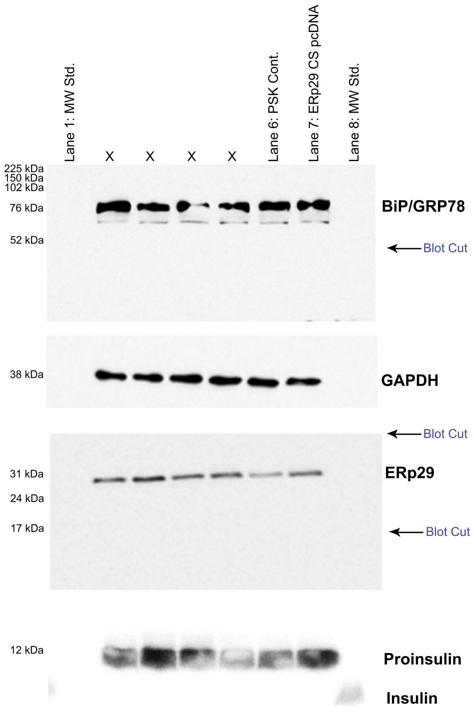

Supplement: S1 Raw images — (PDF) [file pone.0233502.s004.pdf]
